# Antioxidant Activity, Total Phenolics and Flavonoid Contents of some Edible Green Seaweeds from Northern Coasts of the Persian Gulf

**Published:** 2014

**Authors:** Massoumeh Farasat, Ramazan-Ali Khavari-Nejad, Seyed Mohammad Bagher Nabavi, Foroogh Namjooyan

**Affiliations:** a *Department of Biology , Science and Research Branch ,IslamicAzad University,Tehran, Iran.*; b *Department of Marine Biology, University of Marine Sciences and Technology, Khorramshahr, *, Iran; c *Marine Pharmaceutical Research Center, Pharmacognosy Department, School of Pharmacy, Ahvaz Jundishapur University of Medical sciences.*

**Keywords:** Antioxidant activity, Total phenolics, Flavonoid, Seaweeds, *Ulva*

## Abstract

The antioxidant activity, contents of total phenolics and flavonoids were quantified in the methanolic extracts of four *Ulva* species (*Ulva clathrata *(Roth) C.Agardh*, Ulva linza *Linnaeus*, Ulva flexuosa *Wulfen and* Ulva intestinalis *Linnaeus) grown at different parts of northern coasts of the Persian Gulf in south of Iran. The seaweeds were collected from Dayyer, Taheri and Northern Ouli coasts in April 2011. Methanolic extracts of the seaweeds were assessed for their antioxidant activity using DPPH radical scavenging assay and was performed in a microplate reader. All species exhibited a DPPH radical scavenging activity, and among the species, *Ulva clathrata* demonstrated greater antioxidant potential with a low IC_50_ (0.881 mg mL^-1^) in comparison with those of the other species. Also the highest phenolic content (5.080 mg GAE g^-1^) and flavonoid content (33.094 mg RE g^-1^) were observed in *U.clathrata. *Total phenolic and flavonoid contents showed positive correlations with the DPPH radical scavenging activity (p < 0.01) and negative correlations with IC_50_ (p < 0.01).The results suggest that these edible green seaweeds possess antioxidant potential which could be considered for future applications in medicine, dietary supplements ,cosmetics or food industries.

## Introduction

Free radicals have been claimed to play an important role in affecting human health by causing many diseases (*e.g*., heart diseases, cancer, hypertension, diabetes and atherosclerosis). In the past decade, anti-oxidants have shown their relevance in the prevention of various diseases, in which free radicals are implicated (1).

According to the previous studies, terrestrial plants are rich sources of phytochemicals possessing important properties such as antioxidant activity. Many investigators have found several types of anti-oxidants from different parts of various plant species such as oilseeds, cereal crops, vegetables and spices (2). 

Recently, polyphenolic compounds including flavonoids is known as safe and non-toxic anti-oxidants. Many studies have shown that a high dietary intake of natural phenolics is strongly associated with longer life expectancy, reduced risk of developing some chronic diseases, various types of cancer, diabetes, obesity, improved endothelial function and reduced blood pressure (3-5). Phenolic compounds are commonly found in plants and seaweeds. Like other plants, seaweeds contain various inorganic and organic substances, which can benefit human health (6). It has been observed that ROS production in algae is stimulated by various environmental stresses, such as high light levels, heavy metals, high salt concentrations, UV radiation *etc.* Algae generally has higher antioxidant activity due to a higher contents of nonenzymatic antioxidant components, such as ascorbic acid, reduced glutathione, phenols and flavonoids (7). As a result, many marine bio-sources in the last decades have attracted attention in the search for natural bioactive compounds to develop new drugs and healthy foods. Compounds with antioxidant, antiviral, antifungal, antimicrobial, antitumor and anti-inflammatory activities have been found in brown, red and green algae (8).

The antioxidant activity of several seaweeds has been reported (9, 10). *Ulva* genus, an edible seaweed, and an important food source in many south-east Asian countries is also recognized by its synonymous name as *Enteromorpha.* To the best of our knowledge, there is no publication on the antioxidant activities of green seaweeds from Iran. The present study aimed to investigate the antioxidant properties of four *Ulva* species from the northern coasts of the Persian Gulf for future applications in medicine, dietary supplements, cosmetics or food industries. 

## Experimental

*Chemicals*

Ascorbic acid, Folin-ciocalteu reagent, Gallic acid and Methanol were purchased from Merck Company (Darmstadt, Germany). DPPH and Rutin were purchased from Sigma Chemical Co (St.Louis, MO, USA). All the chemicals and reagents used were of analytical grade.

*Collection and preparing of algal extract*

**Figure. 1:**
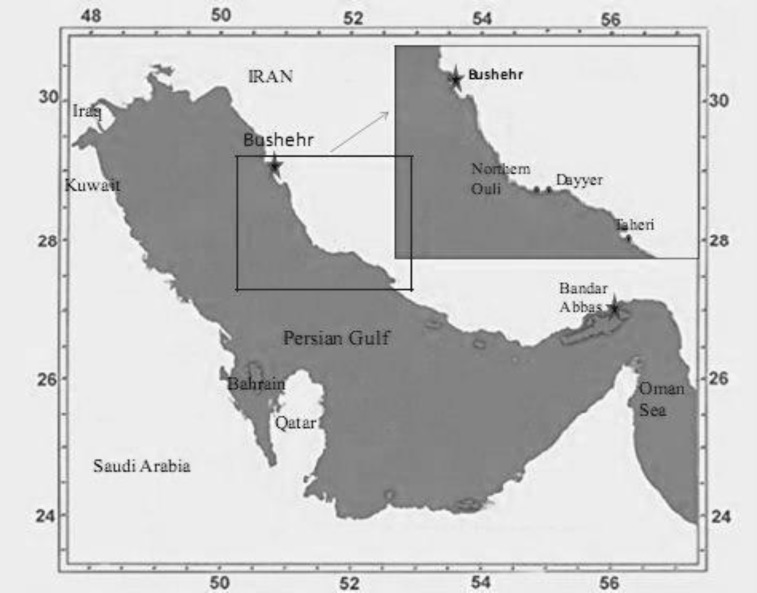
Study area

The seaweeds were collected at low tide time(according to the tide time table obtained from www.iranhydrography.org) along the northern coasts of the Persian Gulf, from Dayyer, Taheri and Northern Ouli (Figure 1) in April 2011. The latitude and longitude of each sampling location was recorded by GPS tracking device.

Once harvested, seaweeds were washed with fresh water to remove sands, salts and epiphytes, and then, were air-dried at room temperature with good controlled air condition carefully. The algae samples were pressed and stored in 5% formol for identification. Voucher specimens were deposited in Jundishapur Marine Pharmaceutical Research Center herbarium. Morphological and anatomical examinations of cell structures were done with the aid of stereomicroscope and light microscope. The samples were identified according to the characteristics and identification keys in the taxonomic publications (11-15). Samples kept at -50 ºC until experiments were processed and milled into powder before extraction.

Dried seaweed sample powder (200 mg) was extracted with 6 mL 80% methanol in an ultrasonic bath for 20 min, vortexed for 30 min and then left to stand at room temperature for 48 h. The extract centrifuged at 1500 g for 10 min, filtered through Watmann No.1 filter paper and then, was freeze dried. The dried extracts were weighed and the yield of each extract was calculated. The stock solutions of the extracts were adjusted with 80% methanol to final concentration of 2 mg (dry extract) mL^-1^. Dilutions were made to obtain concentrations 1, 0.5 and 0.1 mg mL^-1^.

*DPPH free radical scavenging activity*

DPPH radical scavenging activity was determined according to the method of Zhang *et al*. (2007) with slight modifications (16). Briefly, 100 µL of each extract at various dilutions, were mixed with 100 µL of 0.16 mM DPPH solution .The mixture was vortexed for 1 min, kept for 30 min in dark and then, the absorbance was measured at 517 nm in an automated microplate reader (Sunrise-Elisa Reader, Tecan, Swiss). The antioxidant capacity was calculated using the following equation: 

% Inhibition = (A_cotrol_ - (A_sample _- A_blank_) / A_cotrol_) × 100

Where the A_cotrol _is the absorbance of the control (DPPH without sample), the A_sample _is the absorbance of the test sample (the sample test and DPPH solution), and the A_blank _is the absorbance of the sample blank (Sample without the DPPH solution). The half-maximal inhibitory concentration (IC_50_) was calculated by linear regression analysis and expressed as mean of three determinations. Ascorbic acid was used as positive control.

*Determination of total phenolic compounds and flavonoid content*

Total phenolic compounds (TPC) of algal extracts was determined by Folin-Ciocalteu reagent according to the method of Antolovich *et al*. (2002) (17) with minor modifications. In Brief, 20 µL of extracts were mixed with 100 µL of 1:10 Folin-Ciocalteu reagent followed by the addition of Na_2_CO_3 _(80 µL, 7.5%). The assay was carried out in microplate. After incubation at room temperature for 2 hours in dark, the absorbance at 600 nm was recorded. Gallic acid was used as the standard reference. TPC was expressed as mg Gallic acid equivalents per gram of dried extract (mg GAE g^-1^).

Flavonoid content of each extract was determined by following colorimetric method (18). Briefly, 20 µL of each extract were separately mixed with 20 µL of 10 % aluminium chloride, 20 µL of 1 M potassium acetate and 180 µL of distilled water, and left at room temperature for 30 min. The absorbance of the reaction was recorded at 415 nm. The calibration curve was prepared by using Rutin methanolic solutions at concentrations of 12.5 to 100 µg mL^-1^. FC was expressed as mg Rutin equivalents per gram of dried extract (mg RE g^-1^).

*Statistics*

Data were expressed as means ± standard errors of three replicate determinations. All statistics analyses were carried out using SPSS 16.0 for Windows. To determine whether there were any differences among the means, one way analysis (ANOVA) and the Duncan’s new multiple range test were applied to the result. p-values < 0.05 were regarded to be significant. The Pearson correlation analysis was performed between antioxidant activity and total phenolic and flavonoids, and also between total phenolic and flavonoid contents.

## Results and Discussion

*DPPH radical scavenging activity*

During the study, four edible *Ulva* species were collected from northern coasts of the Persian Gulf. *U.intestinalis* collected from two different locations (Dayyer and Northern Ouli). The species, use and medicinal effects of them and their collection information are listed in Tables 1 and 2. The Extraction yields of samples (S1-S5) were 10.60, 28.43, 20.42, 13.39 and 25.82 %, respectively. Due to the presence of different bioactive components with antioxidative potential in the crude extracts of the samples, many different methods have been used to investigate various samples in recent years. In the current study, the DPPH radical scavenging method used to evaluate the antioxidant capacity of the seaweed extracts, because of reliability of the test (19). All seaweed extracts showed antioxidant activity to various degrees (Table 3). Lower IC_50 _value indicates higher antioxidant activity. As shown in Table 3, in comparison to the IC_50_ of ascorbic acid (0.043 ± 0.001 mg ML^-1^) as a standard antioxidant,* U.clathrata *(S1) exhibited a relatively high antioxidant activity with a relatively low IC_50 _( 0.881 ± 0.047 mg mL^-1^) which was significantly different (p < 0.05 ) compared with those of the other species.

**Figure 2:**
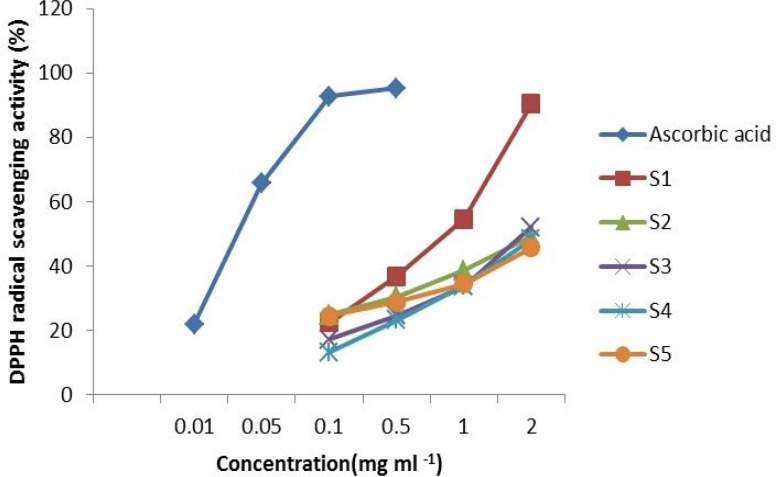
DPPH radical scavenging of algal extracts and Ascorbic acid

The scavenging effect of the tested extracts at concentration of 2 mg mL^-1^ on the DPPH radical decreased in the order of: S1 > S2 > S3 > S4 > S5, and were 90.3, 49.19, 52.15, 48.28 and 45.79% , respectively(Figure 2). The inhibitory effect of all extracts were dose dependent in the range of the tested concentrations. As shown in Figure 2, the inhibitory effect increased with increasing concentration. However, the extract of *U.clathrata *was found to be the most potent scavenger in these tested algae. The activity of the *U.clathrata *extract (2 mg mL^-1^) was comparable to that of the positive control, ascorbic acid (at concentration of 0.1 mg mL^-1^) (p < 0.05). 

**Table 1:** The species, their use and effects

Scientific name	Uses/ medicinal effects
*Ulva clathrata*(Roth) C.Agardh	Anti-tumorigenic, blood anticoagulant activity(35, 36)
*Ulva linza* Linnaeus	Antibacterial and anti-inflammatory activity(37, 38)
*Ulva flexuosa* Wulfen	Cytotoxicity against breast ductal carcinoma cell line, high antibacterial activity(39)
*Ulva intestinalis* Linnaeus	Antibacterial and antihemolytic activities (40)

**Table 2:** The seaweeds and their collection information

Algae	Sample number	Herbarium ID Code	Locality	Latitude, Longitude
***Ulva clathrata***	S1	G110721	Taheri	27º40’04”N- 52º19’71,1”E
***U.intestinalis***	S2	G110421	Dayyer	27º50’01,6”N- 51º56’19,3”E
***U.linza***	S3	G110921	Northern Ouli	27º50’31,6” N- 51º53’08”E
***U.intestinalis***	S4	G110922	Northern Ouli	27º50’31,6” N- 51º53’08”E
***U.flexuosa***	S5	G110923	Northern Ouli	27º50’31,6” N- 51º53’08”E

Many studies have been done to determine antioxidant capacity in *Ulva *species. For instance, 48 marine algae were tested for their antioxidant activity and a low antioxidant activity with a relatively high IC_50 _(43.23 ± 0.28 mg mL^-1^) were reported for *Ulva intestinalis *among the all tested seaweeds (20)*. *However, some researchers have stated high scavenging activity for *Ulva* species. 

For example, three edible species of *Ulva* including *U.compressa, U.* linza and U. tubulosa exhibited high antioxidant activity in linoleic acid system and the best DPPH radical scavenging was observed in methanolic extract of U. compressa (IC_50_ = 1.89 mg mL^-1^) (21). Also, a high value of astaxanthin (a naturally occurring carotenoid pigment and a powerful antioxidant) has been reported in *Ulva intestinalis *(22). It has been shown that, chronic consumption of polysaccharides supplied by *Ulva* species, prevent the fall of antioxidant defences and the development of atherosclerosis in hamsters (23). Besides, some researchers have demonstrated that the natural Ulvan (a group of sulfated heteropolysaccharides obtained from *Ulva* species) and its derivatives exhibited much higher scavenging activity on superoxide radical than vitamin C (24). Moreover, sesquiterpenoids have been isolated from *Ulva fasciata *with free radical scavenging properties (25)*. *Furthermore, Polysaccharides from U. lactuca extract with antioxidant effects in experimentally-induced hypercholesterolemic animal model have been reported (26).

*Total phenolic and flavonoid contents*

Total phenolic content (TPC) and flavonoid content (FC) of the algal extracts are also presented in Table 3. The content of phenolic compounds varied from 5.08 ± 0.65 (*Ulva clathrata*) to 1.258 ± 0.126 (*U.intestinalis* (S5)) mg GAE g^-1^.The phenolic content in the *U.clathrata* extract was significantly different (p < 0.05 ) compared with those of the other species. In general, the higher total phenolic content resulted in higher antioxidant capacity. According to the Table 3, the phenolic content of *U.flexuosa* and *U.intestinalis *(S5) which collected from the same location were significantly different(2.674 ± 0.221 and 1.258 ± 0.126, respectively) (p < 0.05 ) and was higher in *U.flexuosa*. The same result for two *Halimeda* species (of the same area) is repoted by Yoshie *et al*. (2001) (27). This difference in polyphenolic contents may be due to local variations.

As shown in Table 3, the flavonoid content of algal extracts varied from 33.094 ± 2.053 (*Ulva clathrata*) to 8.048 ± 1.119 (*U.intestinalis* (S5)) mg RE g^-1^. The flavonoid contents of two samples of *U.intestinalis* (S3 and S5) were significantly different and were higher in S3 (25.316 ± 2.198 mg RE g^-1^). Despite the fact that, the same species were from the same collection season, however, contents of their flavonoids were different. Previous studies have found marked changes in the chemical constituents with change of seasons and environmental conditions (28).This variation in flavonoid content may be due to the variation in physicochemical parameters such as salinity amongst the selected stations.

**Table 3:** IC_50_ value, TPC and FC of algal extracts

Algae	Sample number	IC_50 _(mg mL^-1^)	TPC(mgGAEg^-1^)	FC (mg RE g^-1^)
***Ulva clathrata***	S1	0.881 ± 0.047 ^a^	5.080 ± 0.650 ^a^	33.094 ± 2.053 ^a^
***U.linza***	S2	1.819 ± 0.632 ^b^	1.996 ± 0.298 ^bc^	10.431 ± 2.215 ^c^
***U.intestinalis***	S3	1.881 ± 0.034 ^b^	1.982 ± 0.308 ^bc^	25.316 ± 2.198 ^b^
***U.flexuosa***	S4	2.175 ± 0.038 ^b^	2.674 ± 0.221 ^b^	9.462 ± 1.558 ^c^
***U.intestinalis***	S5	2.372 ± 0.022^ b^	1.258 ± 0.126 ^c^	8.048 ± 1.119 ^c^

For each treatment the means within the column by different letters are significantly different at P < 0.05

Each value is expressed as the means ±SE (n=3).

The Pearson’s correlation coefficients between the variables are presented in Table 4. As shown in the table, there were strong positive significant correlations between DPPH radical scavenging and contents of phenolics and flavonoids, and high negative correlations between IC_50_ and the variables. Also, the results revealed that there was a strong positive correlation between flavonoids and total phenolics (r = 0.759, p < 0.01). 

**Table 4:** Pearson’s correlation coefficients between the variables

	Phenolic content	Flavonoid content	IC_50_
**Flavonoid content**	0.759**	-	-
**IC**_50_	-0.785**	- 0.804**	-
**DPPH radical scavenging activity**	0.889**	0.819**	-0.866**

** Correlation is significant at the 0.01 level

The antioxidant activity of *Ulva* species were in accordance with their amount of total phenolic and flavonoid contents. Several reports have shown a close relationship between total phenolic content and high antioxidant activity, and many researchers have demonstrated that phenolic compounds are one of the most effective antioxidants in marine algae (29, 30). 

The best-described property of almost every group of flavonoids is their capacity to act as antioxidants. Flavonoids are oxidized by radicals, resulting in a more stable, less-reactive radical. In other words, flavonoids stabilize the reactive oxygen species by reacting with the reactive compound of the radical (31). A positive correlation has been documented between antioxidation capabilities and total polyphenol contents for *Ulva prolifera*, but not with the contents of flavonoids (32). In the current study, strong positive correlations were found between total phenol and flavonoid contents and the antioxidant capacity. Similar observation has been reported by Chai and Wong (2012) (33). The current research findings were in agreement with the results of Bouba *et al*. (2010) which reported a positive correlation between total phenolics and flavonoids in extracts of twenty Cameroonian spices (34). In the current study, only *Ulva clathrata* was collected from middle intertidal rocks where the seaweeds are exposed to UV radiation for several hours in a day. The other tested seaweeds collected from lower intertidal zones. Prolonged seaweed exposure to solar *UV radiation* may result in producing bioactive compounds such as phenolics and flavonoids and may be an explanation of higher antioxidant capacity of *Ulva clathrata* in comparison with the other tested species. 

## Conclusion

In the current study, the antioxidant activities of four *Ulva* species were evaluated. The results clearly indicated that all the tested seaweeds in this investigation possess antioxidant activity. *Ulva clathrata* exhibited high phenolics and flavonoid contents and also, high antioxidant activity with a low IC_50_. Strong positive and significant correlations between DPPH radical scavenging and phenolics and flavonoid contents showed that, phenolic compounds, including flavonoids are the main contributors of antioxidant activity in these *Ulva* species. However, to the best of our knowledge, this is the first report of investigation on the antioxidant capacity and total phenolics as well as flavonoid content of *Ulva* species from Iran. Further work is under way in our laboratories which are aimed at investigation of antioxidant capacity of the other seaweeds of northern coasts of the Persian Gulf and also we are working on the physicochemical parameters of water to find correlation between environment condition and naturally synthesized components by these algae.

## References

[1] Lee HH, Lin CT, Yang LL (2007). Neuroprotection and free radical scavenging effects of Osmanthus fragrans. J Biomed. Sci..

[2] Moraes-de-Souza RA, Oldoni TLC, Regitano-d’Acre MAB, Alencar SM (2008). Antioxidant activity and phenolic composition of herbal infusions consumed in Brazil. Cienc Technol. Aliment..

[3] Halliwell B (2007). Oxidative stress and cancer: have we moved forward?. Biochem J.

[4] Yan S, Asmah R (2010). Comparison of total phenolic contents and antioxidant activities of turmeric leaf, pandan leaf and torch ginger flower. Int Food Res. J..

[5] Jonathan MH, Kevin DC (2006). Dietary flavonoids: Effects on endothelial function and blood pressure. J Sci. Food Agric..

[6] Kuda T, Kunii T, Goto H, Suzuki T, Yano T (2007). Varieties of anti-oxidant and anti-bacterial properties of Ecklonia stolonifera and Ecklonia kurome harvested and processed in the Noto peninsula, Japan. Food Chem.

[7] Wu SC, Wang FJ, Pan CL (2010). The comparison of anti-oxidative properties of seaweed oligosaccharides fermented by two lactic acid bacteria. J Mar. Sci. Technol..

[8] Cox S, Abu-Ghannam N, Gupta S (2010). An assessment of the antioxidant and antimicrobial activity of six edible Irish seaweeds. Int Food Res. J..

[9] Boonchum W, Peerapornpisal Y, Kanjanapothi D, Pekkoh J, Pumas C, Jamjai U, Amornlerdpison D, Noirakasar T, Vacharapiyasophon P (2011). Antioxidant activity of some seaweeds from the Gulf of Thailand. Int J. Agric. Biol..

[10] Saeidnia S, Permeh P, Gohari AR, Mashinchian-Moradi A (2012). Gracilariopsis persica from Persian Gulf contains bioactive sterols. Iran J. Pharm. Res..

[11] BǾrgesen F (1939). Marine Algae from the Iranian Gulf. Danish Scientific Investigation in Iran.

[12] Lawson GW, John DM (1982). The Marine Algae and Coastal Environment of Tropical West Africa, Tropical West Africa. [Beihefte zur Nova Hedwigia 70]. J. Cramer, Vaduz..

[13] Tseng KC (1984). Common Seaweeds of China.

[14] Lewis J, Norris JN (1987). A History and Annotated Account of the Benthic Marine Algae of Taiwan.

[15] Coppejans E, Leliaert F, Dargent O, De Clerck O (2001). Marine algae (Chlorophyta) from the north coast of Papua New Guinea. Cryptogamie Algol.

[16] Zhang ww, Duan XJ, Huang HL, Zhang Y, Wang B G (2007). Evaluation of 28 marine algae fromthe Qingdao coast for antioxidative capacity and determination ofantioxidant efficiency and total phenolic content of fractions and subfractions derived from Symphyocladia latiuscula (Rhodomelaceae). J Appl. Phycol..

[17] Antolovich M, Prenzler PD, Patsalides E, Mc Donald S, Robards K (2002). Methods for testing antioxidant activity. Analyst.

[18] Chang C, Yang M, Wen H, Chern J (2002). Estimation of total flavonoid content in propolis by two complementary colorimetric methods. J Food Drug Anal..

[19] Mohadjerani M (2012). antioxidant activity and total phenolic content of Nerium oleander L. grown in north of Iran. Iran. J. pharm. Res..

[20] Zubia M, Robledo D, Freile-Pelegrin Y (2007). Antioxidant activities in tropical marine macroalgae from the Yucatan Peninsula, Mexico. J Appl. phycol..

[21] Ganesan K, Kumar KS, Rao SPV (2011). Comparative assessment of antioxidant activity in three edible species of green seaweed, Enteromorpha from Okha, Northest coast of India. Innovat Food Sci. Emerg. Tech..

[22] Banerjee K, Ghosh R, Homechaudhuri S, Abhijit M (2009). Biochemical composition of marine macroalgae from Gangetic Delta at the apex of Bay of Bengal. African J Basic Appl. Sci..

[23] Godard M, Decorde K, Ventura E, Soteras G, Baccou JC, Cristol JP, Rouanet JM (2009). Polysaccharides from the green alga Ulva rigida improve the antioxidant status and prevent fatty streak lesions in the high cholesterol fed hamster, an animal model of nutritionally-induced atherosclerosis. Food Chem.

[24] Qi H, Liu X, Ma J, Zhang Q, Li Z (2010). In-vitro antioxidant activity of acetylated derivatives of polysaccharide extracted from Ulva pertusa (Chlorophyta). J Med. Plants Res..

[25] Chakraborty K, Paulraj R (2010). Sesquiterpenoids with free-radical-scavenging properties from marine macroalga Ulva fasciata Dlile. Food Chem.

[26] Hassan S, Abd El-Twab S, Hetta M, Mahmoud B (2011). Improvement of lipid profile and antioxidant of hypercholesterolemic albino rats by polysaccharides extracted from the green alga Ulva lactuca Linnaeus. Saudi J. Biol. Sci..

[27] Yoshie Y, Wang W, Hsieh Y, Suzuki T (2001). Compositional difference of phenolic compounds between two seaweeds, Halimeda spp. J Tokyo Univ. Fish..

[28] Manivannan K, Thirumaran G, Karthikai Devi G, Anantharaman P, Balasubramanian T (2009). Proximate composition of different group of seaweeds from Vedalai coastal waters (Gulf of Mannar): Southest coast of India. Middle East J Sci. Res..

[29] Luo HY, Wang B, Yu CG, Qu YL, Su CL (2010). Evaluation of antioxidant activities of five selected brown seaweeds from China. J Med. Plants Res..

[30] Zakaria NA, Ibrahim D, Sulaiman SF, Supardy A (2011). Assessment of antioxidant activity, total phenolic content and in-vitro toxicity of Malaysian red seaweed, Acanthophora spicifera. J Chem. Pharm. Res..

[31] Nijveldt RJ, van Nood E, van Hoorn DEC, Boelens PG, van Norren K, van Leeuwen PAM (2001). Flavonoids: a review of probable mechanisms of action and potential applications. Am J. Clin. Nutr..

[32] Liu CC, Zhao GL, Li YN, Ding ZP, Liu QG, Li JL (2010). Contribution of phenolics and flavonoids to antioxidant activity of ethanol extract from Eichharnia crassipes. Adv Mater. Res..

[33] Chai TT, Wong FC (2012). Whole-plant profiling of total phenolic and flavonoid contents, antioxidant capacity and nitric oxide scavenging capacity of Turnera subulata. J Med. Plants Res..

[34] Bouba A, Njintang YN, Scher J, Mbofung CMF (2010). Phenolic compounds and radical scavenging potential of twenty Cameroonian spices. Agric Biol. J. North Am..

[35] Tang H, Inoue M, Uzawa Y, Kawamura Y (2004). Anti-tumorigenic components of a seaweed, Enteromorpha clathrata. Biofactors.

[36] Shanmugam M, Ramavat BK, Mody K H, Oza RM, Tewari A (2001). Distribution of heparinoid-active sulphated polysaccharides in some Indian marine green algae. Indian J Mar. Sci..

[37] Sukatar A, Karabay-Yavasoglu NU Ozdemir G, Horzum Z (2006). Antimicrobial activity of volatile component and various extracts of Enteromorpha linza (Linnaeus) J. Agardh from the coast of Izmir, Turkey. Ann. Microbiol..

[38] Khan MNA, Choi JS, Lee MC, Kim E, Nam TJ, Fujii H, Hong YK (2008). Anti-inflammatory activities of methanol extracts from various seaweed species. J Environ. Biol..

[39] Khanavi M, Gheidarloo R, Sadati N, Ardekani MR, Nabavi SM, Tavajohi S, Ostad SN (2012). Cytotoxicity of fucosterol containing fraction of marine algae against breast and colon carcinoma cell line. Pharmacogn Mag..

[40] Soltani S, Ebrahimzadeh MA, Khoshrooei R, Rahmani Z (2012). Antibacterial and antihemolytic activities of Enteromorpha intestinalis in Caspian Sea Coast, Iran. J Med. Plants Res..

